# Comparison of classical, coblation, and combined adenoidectomy techniques in paediatric patients: a single-blind, prospective study

**DOI:** 10.1007/s00405-024-08617-w

**Published:** 2024-04-06

**Authors:** Elvin Alaskarov

**Affiliations:** Department of Otorhinolaryngology, İstanbul Medipol University Health Care Practice and Research Center Esenler Hospital, Istanbul, Turkey

**Keywords:** Coblation, Combined adenoidectomy, Endoscopic adenoidectomy

## Abstract

**Background and objectives:**

Adenoidectomy is one of the most commonly performed surgeries in pediatric otolaryngological practice. This prospective study compared three different adenoidectomy techniques' intra-operative and postoperative outcomes in pediatric patients. The techniques evaluated were classical (blind curettage), coblation, and a combined approach.

**Materials and methods:**

Ninety pediatric patients undergoing adenoidectomy were enrolled in the study. The patients were divided into three groups based on the technique used: Group A, classical adenoidectomy (blind curettage); Group B, coblation adenoidectomy and Group C, combined (blind curettage + coblation) adenoidectomy. The intra-operative time, degree of bleeding, and complications during and after the operations were recorded.

**Results:**

Group A had a significantly shorter operative time than the other groups. However, there was no significant difference in the mean operative time between Groups B and C. The mean amount of intra-operative bleeding differed significantly among the groups. Group B had significantly less bleeding than Group A or Group C. The amount of bleeding also differed significantly between Groups A and C. The postoperative pain scores did not differ significantly among the groups. While complications were infrequent in all groups, Group C did not exhibit a higher complication rate than Groups A and B. The absence of residual or recurrent adenoid tissue in any of the groups during long-term follow-up examinations highlights the effectiveness of all three adenoidectomy techniques in preventing adenoid regrowth.

**Conclusions:**

The combined approach, which was one of the techniques studied, demonstrated an intermediate profile in terms of operative time and intra-operative bleeding compared to the classical and coblation techniques. These findings suggest that this combined approach may be a feasible option for adenoidectomy in pediatric patients, considering its similar low incidence of postoperative complications.

## Introduction

Adenoidectomy is a frequently performed surgical procedure in pediatric otolaryngology to resolve the upper-airway obstruction caused by enlarged adenoidal tissue in children. The cold dissection (curettage) method is widely used globally. The short preparation time, operation duration, and lower costs provide significant advantages over other methods. However, due to the lack of direct visualization, complete mastery of the surgical area is not achieved with this technique [1–3].

Various adenoidectomy techniques have gained popularity and are frequently used, including coblation, laser, suction electrocautery, and microdebridement. These techniques can be performed using endoscopic transnasal or trans-oral approaches, which allow adequate visualization to ensure the complete removal of the adenoidal tissue in a more controlled manner, with no damage to the surrounding structures (e.g., pharyngobasilar fascia, mucosa, or velopharyngeal muscles) [2, 4].

The ideal adenoidectomy technique is expected to have characteristics including minimal intra-operative bleeding, complete excision of the residual lymphoid tissue, short operation time, minimal postoperative pain, rapid healing, a low risk of early and late complications, and cost-effectiveness. Previous studies have discussed the advantages and disadvantages of each method.

This study compared the intra-operative and postoperative outcomes of a combined adenoidectomy technique (classical + coblation) with the classical and coblation adenoidectomy techniques.

## Materials and methods

### Study design

We conducted a single-center prospective randomized study at a reference otorhinolaryngology center—the Ear, Nose, and Throat (ENT) Department of İstanbul Medipol University Health Care Practice and Research Center Esenler Hospital, İstanbul, Turkey.

Ninety children undergoing adenoidectomy between September 2021 and March 2023 were included, with 30 patients in each group.

### Ethics statement and statistical analysis

According to the Helsinki Declaration, ethics approval was obtained from the Institutional Ethics Committee of Istanbul Medipol University before the study commenced. (Decision No: E-10840098-772.02-2876). Informed consent was obtained from the first-degree relatives of the patients included in the study.

Statistical analyses were performed using the SPSS software (SPSS Inc., Chicago, IL, USA), and all data are presented as means ± standard deviations (SD) (range). Student’s *t* test, the Chi-square, and the Mann–Whitney *U* test were used to compare groups (n = 90) in the overall patient evaluation. ANOVA was used for the comparison of independent groups. A *p *value < 0.05 was considered statistically significant.

### Patient selection

Patient selection was based on the children’s medical histories, a physical examination, and radiological findings. The patients and their relatives were unaware of the adenoidectomy technique that would be used. The patients enrolled were 4–12 years old and presented with symptoms of upper-airway obstruction (Table 1).

**Table 1:** Demographic and clinical characteristics of each group of patients

	Group A (Classical) n = 30	Group B (Coblation) n = 30	Group C (Combined) n = 30	P value
Age, years (± SD)	6.51 (± 1.74)	6.54 (± 1.62)	6.15 (± 1.81)	
Males Females	14 (46.6%) 16 (53.3%)	11 (36.6%) 19 (63.3%)	16 (53.3%) 14 (46.6%)	p > 0.05
Adenoid grade (± SD)	3.29 (± 0.46)	3.41 (± 0.50)	3.45 (± 0.50)	

Patients who underwent adenoidectomy together with tonsillectomy or any other surgical procedure were excluded from the study. Our study sample did not include patients with chronic diseases or syndromes.

The physical examination included an evaluation of adenoid hypertrophy, which was assessed using flexible endoscopy. In cases of pediatric noncompliance, septal deviation, or inferior turbinate hypertrophy, lateral airway radiography was requested to assist with grading.

During the pre-operative flexible endoscopic evaluation, the degree of obstruction of the choanae by adenoidal tissue was classified into grades 1–4 according to the McMurray and Clemens grading system. Grade I indicates adenoid tissue filling 1/3 of the vertical height of the choana, Grade II indicates filling up to 2/3, Grade III indicates filling from 2/3 to nearly all but not complete filling of the choana, and Grade IV indicates complete channel obstruction [5].

### Surgical technique

The patients were divided into three groups based on the technique used: Group A (classical adenoidectomy with blind curettage), Group B (endoscopy-assisted coblation adenoidectomy), and Group C (combined adenoidectomy with blind curettage + coblation). All surgeries were performed under general anesthesia by a single fully accredited surgeon.

**Fig. 1:**
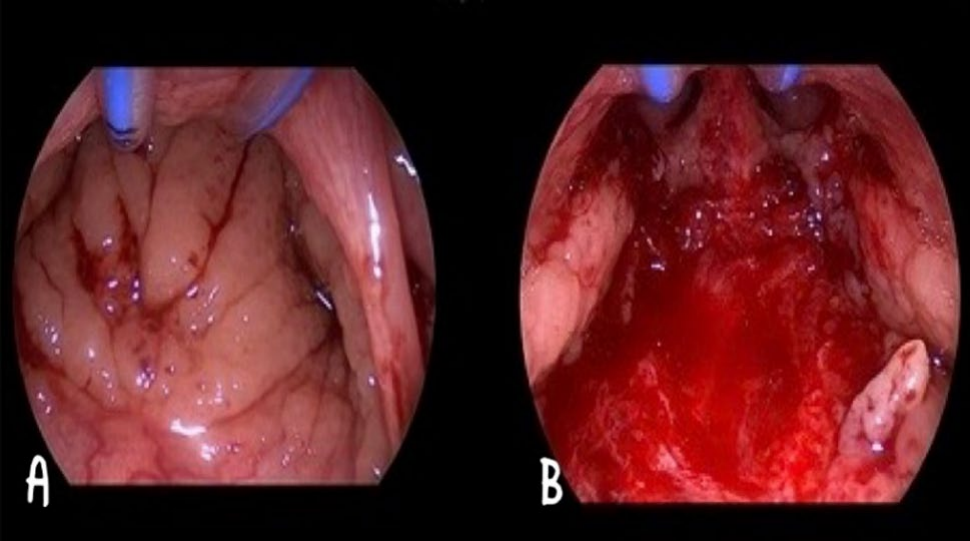
**A** Intra-operative endoscopic view (trans-oral approach) of the hypertrophic adenoid. **B** The final surgical result after classical adenoidectomy

In Group A, sufficient exposure was achieved with a Crowe–Davis mouth gag under general anesthesia. The adenoid tissue was checked with a digital hand, and curettage was performed with an appropriate adenotome. The surgical field was controlled with palpation and an indirect view of the laryngeal mirror to ensure residual adenoid tissue. Temporary packing was applied for hemostasis. The pre-operative hypertrophic adenoid tissue (Fig. 1A) and the surgical field postoperatively (Fig. 1B) of the patients in this group were demonstrated as examples using a 70-degree trans-oral endoscope.

In Groups B and C, under general anesthesia and with sufficient exposure, a blue plastic catheter was passed through both nasal cavities into the nasopharynx and oral cavity, suspending the soft palate.

**Fig. 2:**
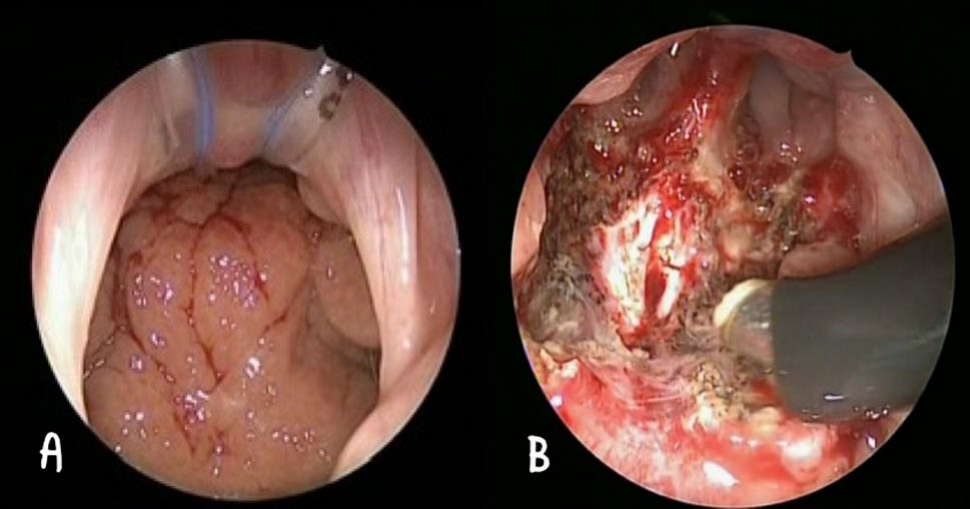
**A** Intra-operative endoscopic view (trans-oral approach) of the hypertrophic adenoid. **B** The final surgical result after coblation adenoidectomy

Endovision was provided using a 70° angle, 4 mm rigid endoscope, which allowed clear visualization of the nasopharynx and choanae (Fig. 2A).

The pathological samples in Group B were excised with a punch under endoscopic guidance. The coblation procedure was performed, then ensuring haemostasis (Fig. 2B), No packing or electrocautery was used.

**Fig. 3:**
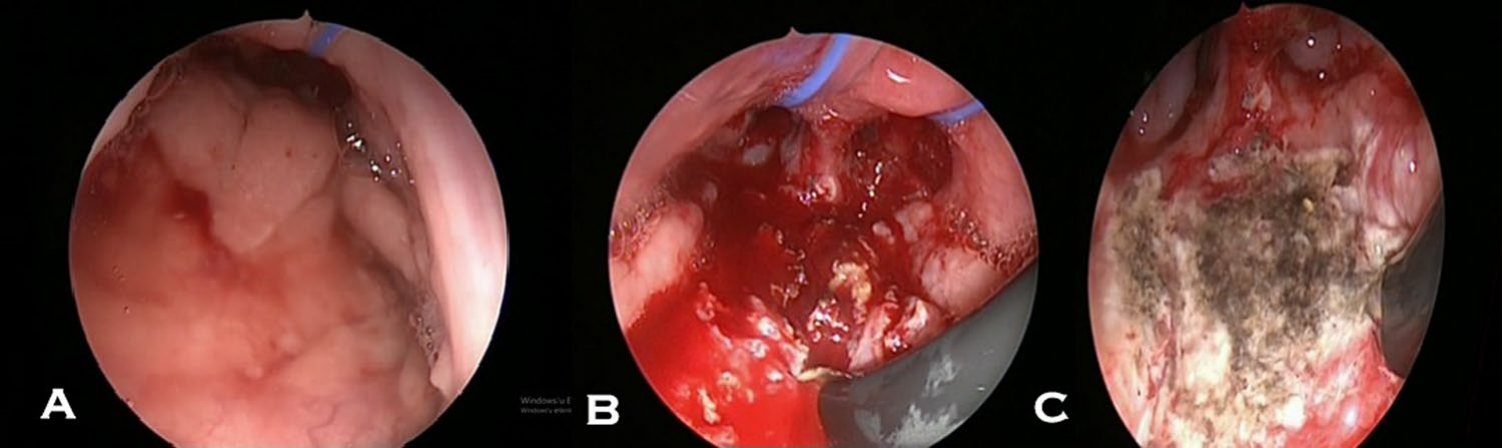
**A** Intra-operative endoscopic view (trans-oral approach) of the hypertrophic adenoid. **B** View of the tissue removed after first curette adenoidectomy. **C** The final surgical result after combined adenoidectomy

In Group C, the adenoid tissue (Fig. 3A), was first removed with curettage (Fig. 3B), and then endoscopy-assisted coblation was used to any residual adenoid tissue and to achieve haemostasis (Fig. 3C).

The intra-operative parameters recorded included the operative time and the degree of bleeding. The operative time was calculated from the start of adenoidectomy until haemostasis was achieved. The amount of bleeding was calculated by subtracting the volume of irrigation fluid used from the total volume of fluid in the aspirator container at the end of surgery.

All patients were monitored at the hospital on the day of surgery, and their pain levels were recorded using the Wong–Baker FACES^®^ Pain Rating Scale after 24 h. The scale contains a series of six faces ranging from a happy face at 0 to indicate “no hurt” to a crying face at 10 to indicate “hurts worst” [6].

Postoperative complications were recorded, such as bleeding, speech impairment, difficulty in oral feeding, re-hospitalization, or re-operation. All patients underwent a postoperative nasopharyngeal examination after 3 and 6 months to detect the presence of any residual or recurrent adenoid tissue.

## Results

The demographic characteristics of the patients enrolled in the study are presented in Table 1. The mean ages in Group A, Group B, and Group C were 6.51 (± 1.74), 6.54 (± 1.62), and 6.15 (± 1.81) years, respectively. No statistically significant difference was observed in the age distribution among the groups (p > 0.05) (Table 1).

According to flexible endoscopic evaluation, the overall average adenoid grade was 3.28, and it did not differ significantly among the groups (p > 0.05).

**Fig. 4:**
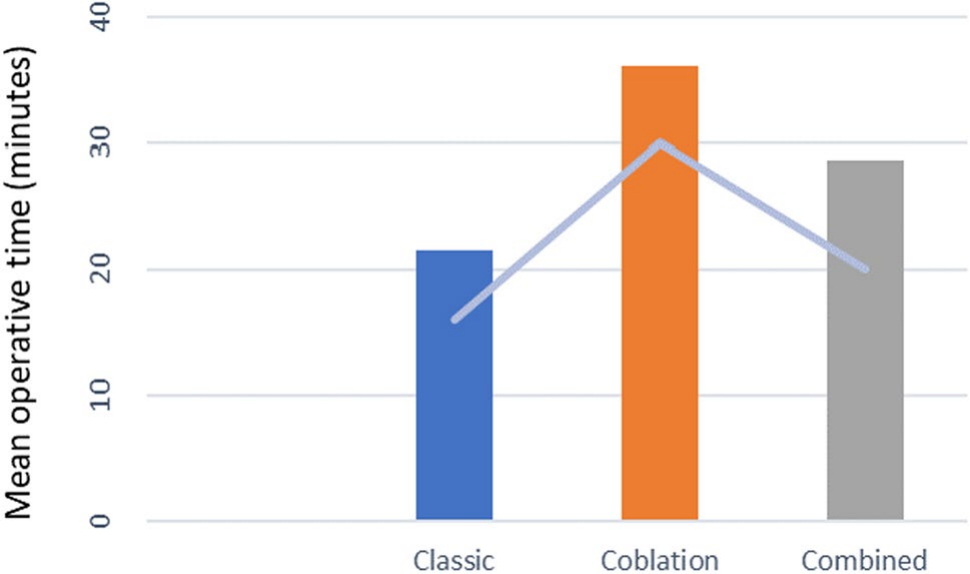
Comparison of mean operative times in all three groups

In terms of intra-operative data, the mean operative time was 21.5 (± 4.7) min in Group A, 36.12 (± 4.82) min in Group B, and 28.67 (± 4.78) min in Group C (Fig. 4). Analysis of variance (ANOVA) revealed a statistically significant difference (F statistic: 29.86, p value = 0.000 at α = 0.05) in operative time among the groups. Group A had a significantly shorter operative time than the other two groups. However, there was no statistically significant difference in the average operative times of Groups B and C (p = 0.150; (Table 2).

**Table 2:** Intra- operative parameters and postoperative pain scores for all three groups

Parameters	Group A (Classical)	Group B (Coblation)	Group C (Combined)	Remarks (p)
Mean operative time, minutes (± SD)	21.5 (± 4.7)	36.12 (± 4.82)	28.67 (± 4.78)	p < 0.05
Intra-operative blood loss, mL (± SD)	24.06 (± 4.32)	11.45 (± 2.31)	16.04 (± 2.23)	p < 0.05
Postoperative pain score (± SD)	4,02 (± 0.79)	3,85 (± 0.45)	3,76 (± 0.58)	Not significant

**Fig. 5:**
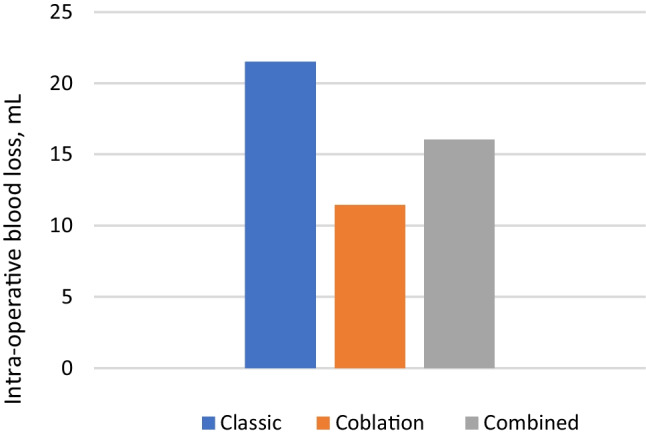
Comparison of mean intra-operative bleeding in all three groups

The mean amount of intra-operative bleeding was 24.06 (± 4.32) mL in Group A, 11.45 (± 2.31) mL in Group B, and 16.04 (± 2.23) mL in Group C (Fig. 5). ANOVA showed statistically significant differences (F statistic: 179.59, p value = 0.000 at α = 0.05) among the groups. Group B had significantly less intra-operative bleeding than the other two groups. The amount of bleeding also differed significantly between Groups A and C (p = 0.000).

No statistically significant difference was observed in the postoperative pain scores among the groups (Table 2).

Two patients in Group A experienced postoperative bleeding within the first 3 days of surgery. Inspection revealed blood clots in their nasopharynges, but no active bleeding was detected. These patients were managed conservatively, and one of them was discharged the following day because they resided far from the hospital.

In Group B, the relatives of one patient sought medical attention at our clinic in the first week after surgery for bad breath and a fever of 38.2 °C in the patient. After examination, oral antibiotic therapy (600 mg penicillin) was initiated, and nasal irrigation was recommended. The patient's symptoms had improved at a follow-up examination 5 days later.

Postoperative voice changes and speech impairment were observed in one child in Group B. During the flexible endoscopic examination, it was observed that the right half of the soft palate was immobile during speech, which led to a preliminary diagnosis of velopharyngeal insufficiency. Speech therapy was recommended, and at the 2-week follow-up, the child’s speech was fully restored, and complete closure of the velopharyngeal opening was observed (Table 3).

**Table 3:** Comparison of complications according to treatment group

	Classical (n = 30)	Coblation (n = 30)	Combined (n = 30)	P value
Infection	0	1	0	
Postoperative bleeding	2	0	0	P > 0.05
Velopharyngeal insufficiency	0	1	0	
Re-hospitalization	1	0	0	
Recurrence	0	0	0	

However, no difference was observed in the complication rate between the study groups (p > 0.05).

No residual or recurrent adenoid tissue was observed in any of the 3 groups in any of the long-term (6 month) control examinations.

## Discussion

Adenoidectomy with or without tonsillectomy is commonly performed in pediatric patients.

The most frequently used traditional curettage technique aims to almost completely remove adenoid tissue using adenoid curettes or adenotomes [5–7].

Previous studies have reported the presence of significant residual tissue after the old curettage technique was used, especially around the Eustachian tube, which can contribute to ongoing middle-ear problems. Uncontrolled adenotome strikes in the peritubal area can also lead to complications, such as mucosal and muscle damage or velopharyngeal insufficiency [8, 9].

Cannon and colleagues conducted an adenoidectomy procedure using forceps under direct vision with an endoscope on a patient with residual adenoid tissue obstructing the nasopharynx several years after the previous conventional adenoidectomy [10].

In the current study, no residual or recurrent adenoid tissue was observed in any of the three groups during the 6-month follow-up period.

In recent times, endoscopy has become popular in ENT practice for both examinations and surgery, with the advantages of three-dimensional visualization and ease of use. Consequently, endoscopy-assisted adenoidectomy has gained in popularity [11, 12].

During adenoidectomy, endoscopy can be used transnasally or transorally. However, transnasal endoscopy may be inadequate or traumatic, especially in young children or in cases of septum deviation [13].

In our study, adenoid hypertrophy was primarily assessed using flexible endoscopy. Additionally, patients in Groups B and C underwent trans-oral visualization during surgery using a 70-degree endoscope.

Coblation adenoidectomy has also been widely performed in recent years. Endoscopy with coblation assistance allows the more-precise excision of peritubal adenoid tissue in the lateral area of the nasopharynx, improving the surgical outcomes in patients with adenoid hypertrophy or OME.

During coblation, maintaining an average temperature of 40–50 °C around the tissue is better than thermal coagulation at 100 °C.Therefore,the risk of tissue necrosis and infection is less anticipated after coblation [14, 15].

In the present study, cautery was never used for haemostasis. However, we observed one case of postoperative infection associated with tissue necrosis in the coblation group.

Previous studies have recorded significantly longer operative times with coblation adenoidectomy than with conventional or microdebridement methods [16–18].

Consistent with the literature, in this study, we also observed longer operative times in the coblation group.

In the study by Pagella and colleagues, a combined technique- the trans-oral endonasal-controlled combined adenoidectomy (TECCA) method was used for adenoidectomy, involving curettage with an adenotome, followed by the excision of the residual tissue with a transnasal microdebrider [19].

Our combined adenoidectomy technique utilizes a hybrid approach to leverage the benefits of both conventional and coblation adenoidectomy techniques. We expected that removing most of the lymphoid tissue with an adenotome would reduce the operative time. Although the hypertrophic adenoidal tissue was reduced when the combined technique was used, the average operative time did not differ significantly from those in the other groups. This may be due to the limited sample size in our study, which may have prevented the detection of such a difference.

The combined technique offers significant advantages over classical adenoidectomy, including effective bleeding control (a significant difference in bleeding was observed between the two techniques) and the prevention of residual tissue in the nasopharynx.

While adenoidectomy is generally a safe and effective procedure, there are potential complications, such as bleeding, infection, and pain. The most common complications, such as bleeding, typically occur in the immediate perioperative period but can develop up to 2 weeks postoperatively [20].

In our study, two Group A patients exhibited inactive bleeding, which were closely monitored without requiring surgical intervention.

Adenoidectomy, when performed in cases of developmental palatal abnormalities such as submucous cleft, has the potential to cause velopharyngeal insufficiency and result in hypernasal speech.

However, careful visualization is required to ensure the preservation of the supporting structure of adenoid tissue for velar closure [21].

In Group B, one patient was suspected of developing velopharyngeal insufficiency during the first week after the surgery. However, after a two-week course of conservative therapy, complete recovery was observed.

Our study had several limitations, including the small number of patients, its performance at a single center, and the short follow-up period. The lack of double blinding was also an important limitation.

Future studies with larger samples and longer follow-up periods are necessary to validate these findings and evaluate additional objective criteria and costs.

Nevertheless, our study is unique because it prospectively evaluated three different groups in a single-blinded manner, and there are no similar studies in the literature. Based on a literature review and our study, future research should include sufficient numbers of patients and utilize a double-blind, randomized prospective design when comparing the three different techniques.

## Conclusions

This single-blind prospective study offers valuable insights into three adenoidectomy techniques.

To improve surgical success, a combined approach that utilizes the strengths of both methods should be considered, if necessary. Therefore, we took advantage of the combined method in the third group of the study. The combined approach has shown advantages in controlling bleeding and removing residual tissue, making it a promising alternative to classical and coblation techniques.

## Data Availability

The patients’ information was taken from the https://mebis.medipol.edu.tr/
